# New agents and regimens for diffuse large B cell lymphoma

**DOI:** 10.1186/s13045-020-01011-z

**Published:** 2020-12-14

**Authors:** Liang Wang, Lin-rong Li, Ken H. Young

**Affiliations:** 1 Department of Hematology, Beijing TongRen Hospital, Capital Medical University, Beijing, 100730 China; 2 Beijing Advanced Innovation Center for Big Data-Based Precision Medicine, Beihang University & Capital Medical University, Beijing TongRen Hospital, Beijing, 100730 China; 3 Peking Union Medical College Hospital, Beijing, 100560 China; 4 Division of Hematopathology, Department of Pathology, Duke University Medical Center and Cancer Institute, Durham, NC 27710 USA

**Keywords:** Diffuse large B cell lymphoma, Chimeric antigen receptor T cells, Immunotherapy, Chemoresistance, Novel agents, Genetic classification

## Abstract

As a widely recognized standard regimen, R-CHOP (rituximab plus cyclophosphamide, doxorubicin, vincristine, and prednisone) is able to cure two-thirds patients with diffuse large B cell lymphoma (DLBCL), and the remaining patients suffer from refractory or relapsed disease due to resistance to R-CHOP and fare poorly. Unsatisfied outcomes for those relapsed/refractory patients prompted efforts to discover new treatment approaches for DLBCL, including chimeric antigen receptor T cells, bispecific T cell engagers, immunomodulatory drugs, immune checkpoint inhibitors, monoclonal antibodies, antibody–drug conjugates, molecular pathway inhibitors, and epigenetic-modifying drugs. Herein, up-to-date data about the most promising treatment approaches for DLBCL are recapitulated, and novel genetic classification systems are introduced to guide individualized treatment for DLBCL.

## Introduction

Diffuse large B cell lymphoma (DLBCL) is the most common subtype of lymphoma in adults worldwide, composing about one-third of non-Hodgkin lymphomas (NHLs) diagnosed each year [1], and it represents a considerable socioeconomic burden affecting millions of people [2]. CHOP (cyclophosphamide, doxorubicin, vincristine, and prednisone) regimen has been used for more than 40 years, and rituximab was approved by the US Food and Drug Administration (FDA) in 2006 for use as first-line treatment of patients with DLBCL in combination with CHOP. Thereafter, R-CHOP regimen has become the standard of care for patients with newly diagnosed DLBCL, even though patients with non-germinal center B cell (non-GCB) subtype of DLBCL have significantly inferior outcomes than their GCB subtype counterparts treated with R-CHOP [3]. In recent years, several randomized clinical trials have been conducted by adding novel targeted agents to R-CHOP (the so-called R-CHOP + X mode) in order to improve outcomes for patients with non-GCB or activated B-cell-like (ABC) subtype of DLBCL, such as bortezomib [4], lenalidomide [5], or ibrutinib [6]. However, none of these targeted agents have been found to confer benefits in these trials. Moreover, dose-adjusted EPOCH (etoposide, prednisone, vincristine, cyclophosphamide, and doxorubicin) plus rituximab (DA-EPOCH-R) also failed to show improvement in survival outcomes for patients with DLBCL in a phase III randomized study (CALGB 50303) [7]. Standard R-CHOP regimen is able to cure two-thirds patients of DLBCL, and the remaining patients suffer from refractory or relapsed disease due to resistance to R-CHOP and fare poorly [8]. The international SCHOLAR-1 study reported the median overall survival (OS) to be only 6.3 months for patients who were refractory to first-line treatment [9].

Poor outcomes for patients who failed R-CHOP regimen prompted efforts to discover new treatment approaches for DLBCL, both up-front and at the time of relapse. With hundreds of clinical trials underway, the landscape for DLBCL treatments has become increasingly crowded. In recent years, several agents or approaches have received the FDA approval for DLBCL, including polatuzumab vedotin, selinexor, tafasitamab, tisagenlecleucel, and axicabtagene ciloleucel (Table 1). Together, the therapeutics targeting immune checkpoints, tumor microenvironment, molecular signaling pathways, and epigenetic aberrations, as well as cellular immunotherapy, constitute the new landscape of treatments for DLBCL. This review focuses on available data about the most promising and potent agents now in clinical testing and provides expertise on individualized treatment for DLBCL according to novel genetic and molecular classifications.

**Table 1:** FDA-approved agents for the treatment of diffuse large B cell lymphoma

Agent	Approved date	Study	Dose schedule^a^	Number of patients^a^	Efficacy^a^
Tafasitamab-cxix (Monjuvi)	July 31, 2020	NCT02399085	12 mg/kg as an intravenous infusion according to the following dosing schedule Cycle 1: Days 1, 4, 8, 15, and 22 of the 28-day cycle Cycles 2 and 3: Days 1, 8, 15, and 22 of each 28-day cycle Cycle 4 and beyond: Days 1 and 15 of each 28-day cycle	80	ORR: 55%; CR: 37%
Selinexor (XPOVIO)	June 22, 2020	NCT02227251	60 mg orally on days 1 and 3 of each week	134	CR: 13%; ORR: 29%
Polatuzumab vedotin-piiq (Polivy)	June 10, 2019	NCT02257567	1.8 mg/kg for six 21-day cycle with bendamustine and a rituximab product	80	CR: 40%; ORR: 63%
Tisagenlecleucel (Kymriah)	May 1, 2018	NCT02445248	0.6–6.0 × 10^8^ CAR-positive viable T cells	68	CR: 32%; ORR: 50%
Axicabtagene ciloleucel (Yescarta)	October 18, 2017	NCT02348216	2.0 × 10^6^ /kg CAR-positive viable T cells (maximum 2 × 10^8^)	108	CR: 51%; ORR: 72%
Hyaluronidase human and rituximab (RITUXAN HYCELA)	June 22, 2017	NCT01649856	1400 mg subcutaneous rituximab and 23,400 units hyaluronidase human, with CHOP	381	CR: 51%; ORR: 83%
Rituximab (Rituxan)	February 10, 2006	LNH 98–5/GELA	rituximab 375 mg/m^2^ with CHOP	399	CR:75%; 2-y OS: 69%
		E4494	rituximab 375 mg/m^2^ with CHOP	632	2-y OS: 74%; PFS: 3.1 years
		MInT	rituximab 375 mg/mm^2^ with CHOP/CHOP-like regimens	823	2-y OS: 95%

*CR* complete response, *ORR* objective response rate, *EFS* event-free survival, *2-y OS* overall survival at 2 years, *PFS* progression-free survival, *CHOP* cyclophosphamide, doxorubicin, vincristine, and prednisone, *CAR* chimeric antigen receptor

^a^Refers to the FDA approval data posted on http://www.fda.gov/drugs

## Immunotherapy

### Chimeric antigen receptor T cells and natural killer (NK) cells

#### Anti-CD19 CAR T cells

**Fig. 1:**
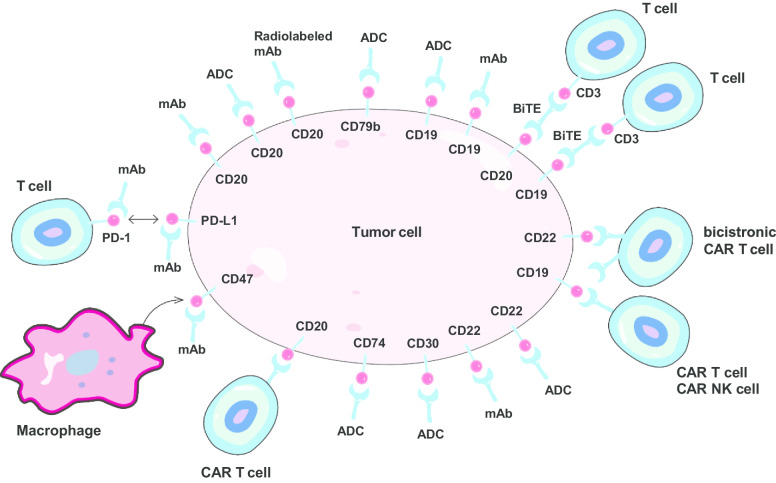
Novel agents and strategies targeting DLBCL cell surface antigens. *mAb* monoclonal antibody, *ADC* antibody–drug conjugate, *BiTE* bispecific T cell engager, *CAR* chimeric antigen receptor, *NK* natural killer, *PD-1* programmed cell death protein 1

**Fig. 2:**
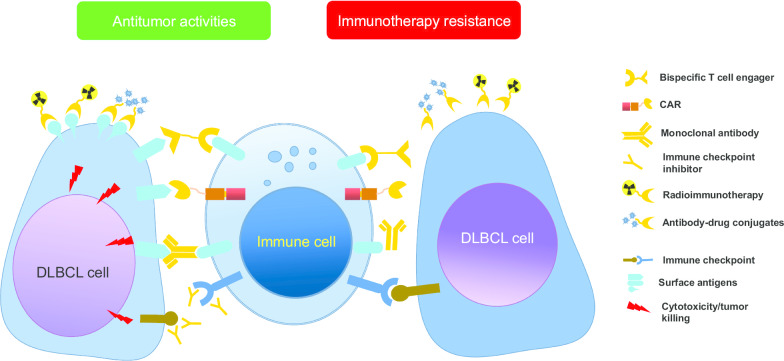
Illustration of antitumor activities of various immunotherapies and potential resistance in DLBCL

Chimeric antigen receptor (CAR) T cells are rapidly emerging as a promising cellular immunotherapy in relapsed/refractory (r/r) DLBCL (Figs. 1, 2). The potent therapeutic efficacy of axicabtagene ciloleucel (axi-cel, marketed as Yescarta) [10], lisocabtagene maraleucel (liso-cel) [11], and tisagenlecleucel (marketed as Kymriah) [12] has been demonstrated in the context of CD19-directed CAR T cell therapy. In ZUMA-1 study, 101 patients of refractory aggressive B cell NHL with a median of three prior lines of treatment received at least 1.0 × 10^6^ CAR-positive T cells/kg, and the investigator-assessed ORR was 83%, and CR rate was 54% [10]. The 2-year follow-up data from ZUMA-1 indicated that axi-cel could obtain durable responses and significantly improve the OS with a manageable long-term safety profile in patients with r/r DLBCL [13]. Another 93 patients who were ineligible or had disease progression after ASCT received tisagenlecleucel, and the best ORR was 52%, with a CR rate of 40%. At 1 year after initial response, the estimated relapse-free survival rate was 65%, indicating a durable response with tisagenlecleucel [12]. Compared with historic data, these CAR T cell products have offered unexpected durable responses in patients with heavily pretreated DLBCL [14], which promoted the approval of Yescarta and Kymriah by FDA. With improved access to these CAR T cell products, patients of r/r DLBCL may be treated with CAR T cell therapy at second-line scenario, or even as first-line treatment for patients with double-hit lymphoma.

#### Dual CAR T cells or combination with immune checkpoint inhibitors

However, despite notable clinical responses, modest durability of responses, treatment-related toxicities, and time-consuming production are major obstacles limiting the clinical use of autologous CAR T cell therapy. Relapses after CD19 CAR T cell therapies are partially due to CD19 loss (Fig. 2) or programmed death ligand 1 (PD-L1) upregulation [15, 16]. In this regard, CAR T cells engineered to secrete human anti-PD-L1 antibodies, and dual CAR T cells as well as incorporation of immune checkpoint inhibitors are considered. For the treatment of B cell lymphomas, dual CAR T cells targeting CD19 and CD20 or CD22 are appealing. In a phase 1 trial, a bispecific CAR T product targeting CD19 and CD22 (Fig. 1) achieved 60% ORR in 5 patients with r/r DLBCL (1 CR and 2 PR) with tolerable toxicities [17]. Moreover, combination of anti-CD19 and anti-CD20 CAR T cells achieved an overall response rate (ORR) of 81.0% and CR rate of 52.4% in 21 patients with r/r DLBCL [18]. It was reported that armed CAR T cells empowered to secrete anti-PD-L1 antibodies could resist T cell exhaustion and improve efficacy against renal cell carcinoma in mice model [19]. Programmed cell death protein 1 (PD1) blockade with pembrolizumab was safe and efficient in some patients with DLBCL progression after CD19 CAR T cell therapy [20]. The first bicistronic anti-CD19/CD22 CAR T cells AUTO3 followed by pembrolizumab showed acceptable safety profiles in a phase 1/2 trial. In patients who received at least 150 × 10^6^ CAR T cells and pembrolizumab at day-1 (*n* = 8), the ORR was 75%, with a CR rate of 63% [21]. Moreover, axi-cel in combination with PD-L1 blockade by atezolizumab showed manageable safety profiles and preliminary efficacy, according to the result of ZUMA-6 [22]. Thus, combination of CAR T cell therapy and PD-1/PD-L1 blockade seems feasible and promising in the treatment of r/r-DLBCL (Table 2).

**Table 2:** Summary of CAR T cell results in diffuse large B cell lymphoma

Target	Agent	Study	Study phase	Number of DLBCL patients (treated)	Dose	ORR (%)	CR (%)	References
CD19	Axicabtagene ciloleucel	ZUMA-1 (NCT02348216)	1/2	101	2.0 × 10^6^ CAR T cells/kg	83	58	[13]
CD19	axicabtagene ciloleucel (in combination with atezolizumab)	ZUMA-6 (NCT02926833)	1	12	2.0 × 10^6^ CAR T cells/kg	90	60	[22]
CD19	tisagenlecleucel	JULIET (NCT02445248)	2	93	0.1–6 × 10^8^ CAR T cells	52	40	[12]
CD19	lisocabtagene maraleucel	TRANSCEND NHL 001 (NCT02631044)	1	268	50–150 × 10^6^ CAR T cells	73	53	[139]
CD19	CTL019	NCT02030834	2a	28	1.79–5.00 × 10^6^ CAR T cells	64	43	[11]
CD19	ET019003	NCT04014894	1	6	2–3 × 10^6^ CAR T cells/kg	100	–	[140]
CD19	FMC63-28Z	NCT00924326	1/2	7	1–5 × 10^6^ CAR T cells/kg	85	71	[141]
CD19/CD22	AUTO3 (in combination with pembrolizumab)	ALEXANDER (NCT03287817)	1/2	24	50 × 10^6^ CAR T cells	57	29	[21]

*DLBCL* diffuse large B cell lymphoma, *CAR* chimeric antigen receptor, *ORR* overall response rate, *CR* complete response

#### Universal CAR T cells

Considering the frail condition of some patients and deficient T cell functions due to previous heavy treatments, clinical trials exploring CAR T cell therapy in the first-line (NCT03761056, ZUMA-12) and second-line settings (NCT03391466, NCT04161118, NCT03570892, NCT03575351, NCT03483103) are underway. Furthermore, allogeneic anti-CD19 CAR T cells from healthy donors are recognized to be an effective alternative to patients’ exhausted T cells, as long as the endogenous TCR on the allogeneic CAR T cells is edited through various gene editing technologies in order to avoid alloreactivity of donor-derived T cells. A universal CAR T cell product targeting CD19 (UCART19) has been developed to treat r/r B cell acute lymphoblastic leukemia with promising efficacy and manageable toxicities [23]. Some other products with similar construct to UCART19 are under investigation in the treatment of r/r DLBCL (NCT03939026) (Table 3).

**Table 3:** Summary of antibody–drug conjugates and bispecific antibodies results in diffuse large B cell lymphoma

Target	Drug	Toxin	Combined agents	Study	Study phase	No.	Efficacy	References
CD19/CD3	Blinatumomab	–	R-chemotherapy	NCT 03023878	2	30	ORR 89%	[27]
CD19	Coltuximab ravtansine	DM4	–	NCT 01472887	2	61	ORR 44% CR 15%	[71]
CD19	Loncastuximab tesirine	SG3199	–	NCT 02669017	1	63	ORR 55% CR 37%	[72]
CD20	MT-3724	SLTA	–	NCT 02361346	1	13	ORR 30% CR 10%	[57]
CD20	Ibritumomab tiuxetan	Yttrium-90	Combined with R as maintenance therapy	NCT 00070018	2	33	5-y OS 87% 5-y PFS 82%	[59]
CD20	Tositumomab	Iodine-131	R-CHOP	NCT 00107380	2	86	ORR 86% CR 61% 2-yPFS69% 2-y OS 77%	[61]
CD20/CD3	RG6026	–	Obinutuzumab	NCT 03075696	1b	28	ORR 48% CR 43%	[30]
CD20/CD3	Mosunetuzumab	–	–	NCT 02500407	1/1b	55	ORR 33% CR 21%	[31]
CD20/CD3	REGN1979	–	–	NCT 02290951	1	53	ORR 33% CR 18%	[32]
CD22	Pinatuzumab vedotin	MMAE	Rituximab	NCT 01691898	2	42	ORR 60% CR 26%	[65]
CD22	Inotuzumab ozogamicin	Calicheamicin	Rituximab	NCT 00299494	1/2	42	ORR 74% 2-y EFS 42%	[76]
CD22	Epratuzumab tetraxetan	Yttrium-90	R-CHOP	NCT 00906841	2	71	2-y EFS 75%	[142]
CD30	Brentuximab vedotin	MMAE	–	NCT 02280785	2	12	CR 17% DCR 50%	[78]
CD30	Brentuximab vedotin	MMAE	–	NCT 01421667	2	49	ORR 44% CR 17% mPFS: 4 m	[80]
CD74	STRO-001	Maytansinoid warhead	–	NCT 03424603	1	4	ORR 50% CR 25%	[83]
CD79b	Polatuzumab vedotin	MMAE	Rituximab	NCT 01691898	2	39	ORR 54% CR 21% mDoR 13.4 m	[65]
CD79b	Polatuzumab vedotin	MMAE	R-CHP/GHP	NCT 01992653	1b/2	66	ORR 89% CR 77%	[64]

*MMAE* monomethyl auristatin E, *AEs* adverse events, *NEs* neurologic events, *SLTA* Shiga-like toxin-I A1, *NHL* non-Hodgkin lymphoma, *CRS* cytokine release syndrome, *mDoR* median duration of response, *EFS* event-free survival, *R-CHOP* rituximab, cyclophosphamide, doxorubicin, vincristine, and prednisone, *ORR* objective response rate, *CR* complete response, *GHP* obinutuzumab, doxorubicin, prednisone

#### CAR-NK cells

Similarly, genetically modified allogeneic NK cells represent another promising alternative for CAR T cell therapies. According to data from a phase 1/2 trial, NK cells expressing anti-CD19 CAR and interleukin-15 (Fig. 1) resulted in responses in 73% (8/11) patients, of whom 4 with lymphoma and 3 with chronic lymphocytic leukemia had a CR. The responses were rapid without development of cytokine release syndrome (CRS), neurotoxicity, or graft-versus-host disease, and there was no increase in the levels of inflammatory cytokines, including interleukin-6, over baseline. Of note, the infused CAR-NK cells expanded and persisted at low levels for at least 1 year after infusion [24]. Thus, the HLA-mismatched NK cells originating from an allogeneic source may enable streamlining of the production process and universal access [24].

#### CAR T cells with safety switches

Management of toxicity while maintaining efficacy is a pivotal focus for CAR T cell therapies in development. Nearly half of the patients treated with axi-cel suffered from grade 3 or worse serious adverse events, including CRS and neurotoxicity [13]. The fourth-generation CAR T cells usually contain additional safety measures, such as suicide genes (herpes simplex virus thymidine kinase, or caspase-9) or expression of cell surface antigens that can be targeted by monoclonal antibodies [25]. Moreover, a recombinant antibody-based bifunctional switch could be engineered to consist of a tumor antigen-specific Fab molecule at the one end and a peptide neo-epitope (PNE) at the other end, which can be bound exclusively by a PNE-specific switchable CAR T cell [26]. These types of CAR T cells are active to kill tumors only when they are given concurrently with the specific bifunctional switches, which make both the efficacy and toxicities of CAR T cells controllable.

Overall, diverse CAR T or NK cell products with different targets, different combinations, or different origins are enriching our arsenal in treating r/r-DLBCL, which may be put forward to second-line, or even first-line treatment for high-risk patients in the near future.

### Bispecific T cell engagers

Bispecific T cell engagers (BiTEs, Figs. 1, 2) are a new class of immunotherapy, which enhances the patients’ immune cells to attack tumors by retargeting T cells to tumor cells. Blinatumomab, a CD19/CD3 BiTE, has demonstrated impressive efficacy against B cell acute lymphoblastic leukemia (ALL), which led to its approval by FDA to treat r/r B-ALL. A phase 2 study evaluated the use of blinatumomab following rituximab-based immunochemotherapy in patients with newly diagnosed high-risk DLBCL (*n* = 28), and ORR was reported to be 89% [27]. Blinatumomab enabled 4 patients with no metabolic response after rituximab-based therapy to get objective responses after blinatumomab treatment, and minimal residual disease (MRD, assessed by plasma cell-free circulating tumor DNA) was converted from positive to negative in 9 patients following blinatumomab treatment, indicating blinatumomab consolidation as a potential option for newly diagnosed high-risk DLBCL [27]. In a phase 2 study, blinatumomab was used as second salvage in 41 patients with aggressive B cell lymphoma who failed platinum-based first salvage regimens, and got an ORR of 37% and CR rate of 22% after 12 weeks, indicating blinatumomab monotherapy to be an effective therapy that could bridge autologous stem cell transplantation (ASCT) in r/r aggressive B cell lymphomas [28]. To further improve the efficacy of blinatumomab, combination with immunotherapy agents or immunomodulatory drugs to enhance the anticancer activity of host T cells is under investigation. Phase 1 studies with blinatumomab and pembrolizumab (NCT03340766) or lenalidomide (NCT02568553) are ongoing for patients with r/r DLBCL. However, due to the short half-life of 2–4 h of blinatumomab, continuous intravenous infusion should be administrated for up to 28–70 days, which makes it extremely inconvenient in routine clinical practice. To extend the half-life and allow for a more convenient administration, a next-generation BiTE antibody construct-designated CD19 HLE BiTE (such as AMG 562) has been generated, with a half-life of about 210 h, which enables once-weekly dosing [29]. The preclinical results of AMG 562 have demonstrated similar activity to blinatumomab, and it is now tested in clinical trials enrolling patients of DLBCL, mantle cell lymphoma, and follicular lymphoma (NCT03571828). Glofitamab, mosunetuzumab, and REGN1979 are all CD20/CD3 BiTEs with different construction that proved to be effective in r/r DLBCL. Glofitamab (RG6026), a novel 2-to-1 format BiTE with 2 CD20-binding molecules and 1 CD3-binding molecule, demonstrated higher potency in vitro comparing to other CD20/CD3-BiTEs. In a phase 1 dose-escalating study (NCT03075696), a CR rate of 34.1% and ORR of 49.4% were achieved in 85 patients with aggressive B cell lymphoma who received the dosage of at least 10 mg of glofitamab. Of note, more than half of the patients developed CRS and 16.7% of patients received tocilizumab to control CRS. Concurrent CD20 targeting by glofitamab and obinutuzumab led to an ORR of 48% and CR rate of 43% in r/r aggressive NHL (including DLBCL) in a phase 1b study [30]. A trial investigating the efficacy and safety of combined glofitamab and R-CHOP or G-CHOP is underway in untreated DLBCL (NCT03467373). According to results from a phase 1/1b trial, patients with r/r DLBCL treated with mosunetuzumab had an ORR of 33% and CR rate of 21%. All patients with CR remained in remission at a median follow-up of 372 days [31]. REGN1979 monotherapy at dose 80 mg to 320 mg achieved CR in 5 of 8 patients with DLBCL, including 2 with CAR T cells failure [32]. Thus, BiTEs targeting CD3 and B cell surface antigens, such as CD19 and CD20, provide promising efficacy and tolerable safety profiles. Though not as potent as anti-CD19 CAR T cells, those BiTEs have the advantage of off-the-shelf availability, and serious adverse events could be easily controlled by discontinuing the drug. Future studies should be done concerning the optimal combination therapies and role of BiTEs in various settings of the disease, such as first-line induction, consolidation for high risk.

### Immunomodulatory drugs

Lenalidomide, as an immunomodulatory agent, is proved to have a variety of effects on the immune system and also alter tumor microenvironment by affecting the production and activity of cytokines involved in the maintenance of tumor growth and survival. Meanwhile, lenalidomide could exert direct tumor toxicities via binding to cereblon to inhibit downstream NF-κB signaling [33]. Combination of lenalidomide and R-CHOP21 (R2-CHOP) seemed to provide benefits in several phase 2 studies, especially for the non-GCB and high-risk subgroups [34]. In REMARC study, for elderly patients responding to first-line R-CHOP, lenalidomide maintenance for 24 months prolonged PFS over placebo, although no OS benefit was found [35]. However, the phase 3 ROBUST study in untreated ABC-DLBCL did not meet the primary endpoint of PFS, though positive PFS trends favoring R2-CHOP21 were observed in those with high international prognostic index (IPI) scores and advanced disease stages [36]. Similarly, data from a phase 3 study of lenalidomide and R-miniCHOP showed no outcome improvement for patients aged over 80 years [37]. Meanwhile, the ECOG-ACRINI412 study achieved its primary endpoint, demonstrating significantly better PFS when using R-CHOP21 combined with lenalidomide [38]. Possible explanations for the different trial outcomes may include the different dose (lenalidomide 15 mg d1–14 in ROBUST and 25 mg d1–10 in ECOG-ACRIN 1412), eligibility criteria (exclusively ABC subtype in ROBUST, and both ABC and GCB in ECOG-ACRIN 1412), and time to treatment (within 31 days of diagnosis in ROBUST and within 21 days in ECOG-ACRIN 1412), which indicates that the use of lenalidomide should not be restrained to ABC-DLBCL and timely treatment may benefit patients further for this aggressive lymphoma [38]. Notwithstanding, lenalidomide has been demonstrated to be effective in r/r DLBCL as monotherapy [39] or combining salvage chemotherapies, such as R-ICE [40] and R-ESHAP [41]. The chemo-free regimen R2 (rituximab plus lenalidomide) has also been shown to be active in elderly r/r DLBCL patients, and durable CR was achieved in 35% patients [42], which made R2 an appealing choice for those ASCT-ineligible patients. Moreover, due to the ability of penetrating blood–brain barrier, lenalidomide has been proved to be highly active in treating primary central nervous system (CNS) lymphoma (PCNSL) [43, 44]. Thus, addition of lenalidomide to immunochemotherapy may reduce the risk of CNS relapses, which needs to be validated in the future.

### Immune checkpoint inhibitors (ICIs)

**Fig. 3:**
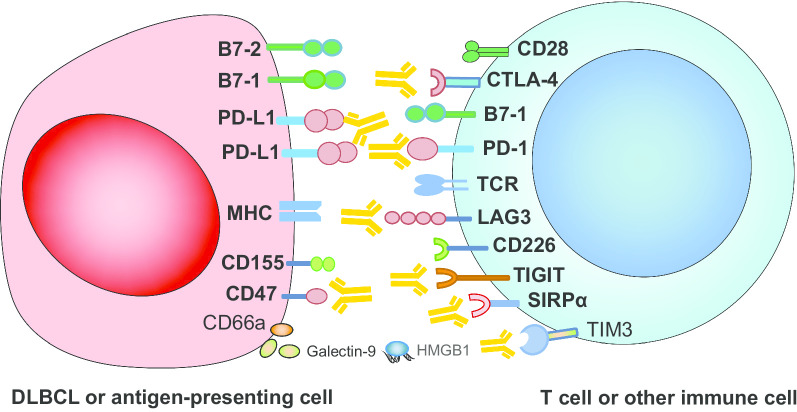
Established and emerging immune checkpoint targets in DLBCL and corresponding blocking monoclonal antibodies as immune checkpoint inhibitors

Immune evasion is a hallmark of DLBCL, where the B7-CD28 gene family plays a pivotal role. According to the data based on a total of 184 DLBCL biopsies, PD-1 (CD279) and PD-L1 (CD273, B7-DC) expressions (i.e., expressed on more than 5% of cells) on lymphoma cells were detected in 1.63% and 43.48% of patients, respectively, while their expressions on microenvironment cells were found in 11.41% and 26.09% of patients, respectively [45]. Several early phase trials are reported, testing multiple inhibitors targeting the most studied immune checkpoints both in the r/r and in the frontline settings, including pembrolizumab and nivolumab for PD-1, durvalumab, avelumab, and atezolizumab for PD-L1 (Fig. 1) [46]. However, only avelumab underwent phase 3 trial for combination therapies in r/r DLBCL (NCT02951156), and no data from phase 3 trials are currently available. Apart from PD-1 and PD-L1, other molecular targets for novel immune checkpoint inhibitors (LAG-3, TIGIT, TIM-3, and VISTA) have been discovered continuously, but remained to be tested in DLBCL (Fig. 3) [47].

PD-1 blockade has been tested in r/r, post-ASCT consolidation, and first-line settings, either monotherapy or in combination mode. Nivolumab as monotherapy at dose 3 mg/kg showed an ORR rate of 36% and acceptable safety profiles in heavily pretreated patients with r/r DLCBL (*n* = 11) [48]. An interesting use of checkpoint inhibition has evolved with the introduction of CAR T cell therapy. As aforementioned, atezolizumab or pembrolizumab following CD19 CAR T cell therapies was expected to tackle resistance, though further research is needed. Also, PD-1 blockade after ASCT was believed to leverage immune landscapes to decrease minimal residual disease. However, data from a phase 2 study showed that pembrolizumab consolidation given after ASCT did not improve the 18-month PFS rate (59%) [49]. Just as rituximab does not provide clinical benefits when used as post-ASCT maintenance, checkpoint inhibitors face the same challenge. Post hoc analysis needs to be done to explore the specific subpopulation who benefited from ICIs therapy, such as PD-L1 amplification or mutation, etc. Thus, immunotherapy targeting PD-1/PD-L1 seems unsatisfactory (Fig. 2) when using as monotherapy, and indicative biomarkers should be explored further to launch precision medicine in a subset of DLBCL patients. Combination of pembrolizumab and R-CHOP (PR-CHOP) has been tested in 30 newly diagnosed DLBCL patients, resulting in the overall and complete response rate being 90% and 77%, respectively. The 2-year PFS was 83% at a median follow-up of 25.5 months, and this regimen was well tolerated [50]. Meanwhile, higher expression of PD-L1 correlated with improved PFS, suggesting assessment of PD-L1 expression as a useful biomarker to identify patients who actually benefit from this first-line strategy.

Though PD1/PD-L1 blockade seems to have unimpressive efficacy in r/r DLBCL, another immune checkpoint CD47, considered as macrophage checkpoint, has emerged to be a promising target (Fig. 1). CD47 upregulation on malignant cells reveals immune evasion and drug resistance, which was detected in 53.7% patients of DLBCL [51]. Hu5F9-G4 is a first-in-class CD47-directed monoclonal antibody (mAb) and macrophage checkpoint inhibitor that preferentially enables phagocytosis of DLBCL cells by CD47 blockade. This action could be augmented by rituximab through its Fc region [51]. The safety and efficacy profiles of the combination of Hu5F9-G4 and rituximab were evaluated in a phase 1b/2 study involving 63 patients with r/r DLBCL. ORR was obtained by 39% (*n* = 18) patients, and 20% (*n* = 9) experienced CR. Duration of response was not reached at more than 20 months of follow-up. Adverse events were mostly grade 1 to 2 infusion reactions (38%) and headache (34%), whereas first-dose grade 3 anemia in 15% of patients was observed [52]. Moreover, dual blockade of CD47 and PD-L1 may be a potential synergistic therapy that can elicit both innate and adaptive immune response against tumors [53], which is worthy investigating in clinical trials (NCT04328831).

## Monoclonal antibodies and antibody–drug conjugates

Since the approval of rituximab in the treatment of DLBCL in 2006, many novel agents targeting cell surface antigens have been developed and tested in DLBCL. Many mAbs are developed both in the unconjugated form and in the conjugated forms (Figs. 1, 2), where they are designed to conjugate to a cytotoxic payload (antibody–drug conjugate, ADC), a radioactive molecule (radiolabeled mAb), or another antibody (i.e., bispecific antibody) by a covalent linker. In the following, these novel antibodies are categorized according to different cell surface antigens (Fig. 1).

### CD20-directed agents

In addition to first-generation rituximab, other CD20 mAbs currently used in the treatment of DLBCL include second-generation ofatumumab as well as third-generation obinutuzumab (GA-101). The alteration within molecular structures (e.g., Fc region) of CD20 mAbs enhanced binding affinity to CD20 antigen and antibody-dependent cell cytotoxicity (ADCC) [54]. However, G-CHOP (obinutuzumab plus CHOP) did not significantly improve PFS but resulted in more severe adverse events, compared with R-CHOP in previously untreated DLBCL [54]. Ofatumumab was well tolerated in the elderly, and combination of ofatumumab and miniCHOP was reported to achieve a 2-year OS rate of 64.7% for DLBCL in patients aged 80 years or older [55]. Meanwhile, for those frail elderly patients who are poor candidates for R-CHOP chemotherapy, combination of ofatumumab and bendamustine demonstrated an ORR of 90.5% and CR of 33.3% with tolerable toxicities [56]. Overall, comparing with historic data of rituximab-based therapy, all these second-generation CD20 mAbs did not provide further benefits for DLBCL patients, and future patient resources should be put in clinical trials of CD20-ADCs instead of CD20-mAbs.

MT-3724 is a novel ADC directed against CD20, which is comprised of a single-chain variable fragment lined to Shiga-like toxin-1A, a ribosome-inactivating protein. Phase I trial of MT-3724 monotherapy in heavily pretreated DLBCL patients reported an ORR of 30% [57], with the phase II trial already being underway [58]. Radiolabeled CD20 mAbs currently used in clinical testing include ibritumomab tiuxetan (Zevalin) and tositumomab (Bexxar), chelated with yttrium-90 and iodine-131, respectively. Consolidation with Zevalin after CHOP plus radiotherapy achieved 5-year OS of 87% and 5-year PFS of 82% in high-risk patients with early-stage NHL including DLBCL [59]. For patients with limited-stage DLBCL who were interim-PET positive after 3 cycles of R-CHOP, involved-field radiation therapy (IFRT) followed by Zevalin consolidation resulted in 5-year PFS rate of 86% and OS rate of 93% in the S1001 study [60]. In the SWOG S0433 trial involving 84 patients with advanced-stage DLBCL, R-CHOP followed by Bexxar consolidation showed a 2-year PFS of 69% and 2-year OS of 77% [61], indicating that consolidation therapy with those radiolabeled CD20 mAbs may provide benefits to patients of high-risk or advanced disease.

### CD79b-directed agents

CD79b, a core component of the B cell receptor, plays a pivotal role in chronic-active B cell receptor (BCR) signaling and canonical NF-κB signaling pathway of DLBCL survival, especially for the activated B cell-like (ABC) subtype [62]. Polatuzumab vedotin (DCDS4501A) is a novel CD79b-directed ADC with site-specific conjugation to MMAE. In 2019, combined polatuzumab vedotin with bendamustine and rituximab was approved by the FDA for patients with r/r DLBCL after at least 2 prior therapies [63]. Beyond combination with rituximab, the replacement of vincristine with polatuzumab vedotin was tested in a multicenter phase Ib/II study with R-CHP or G (obinutuzumab)-CHP. Polatuzumab vedotin dosed 1.8 mg/kg showed overall acceptable safety profiles with 25/66 (38%) patients experiencing grades 1 and 2 peripheral neuropathy and good efficacy (ORR 89%; CR, 77%) in previously untreated DLBCL [64]. Though ITAM (immunoreceptor tyrosine-based activation motif) mutation of CD79b was frequently recognized in 23% of ABC r/r DLBCL [62], the reported activities of polatuzumab vedotin showed no preference for any DLBCL cell-of-origin subtypes or CD79b expression [64, 65]. Giving the significant clinical activities and manageable safety profiles of polatuzumab vedotin, additional evaluation of polatuzumab vedotin with other agents (including lenalidomide, venetoclax, and obinutuzumab) in the r/r setting is ongoing. Specifically, two phase III studies are now recruiting: POLARIX to compare polatuzumab vedotin plus R-CHP with R-CHOP alone in untreated DLBCL [66], and POLARGO evaluating polatuzumab vedotin in combination with R-GemOx (rituximab, gemcitabine, and oxaliplatin) in patients with r/r DLBCL after at least 1 prior therapies [67].

### CD19-directed agents

Recent results of the CD19 mAbs suggest that this therapeutic paradigm is finally showing promise for DLBCL. On July 31, 2020, the FDA approved the use of an Fc-engineered CD19 mAb tafasitamab (MOR208, Monjuvi^®^) combined with lenalidomide in r/r DLBCL. In a phase IIa study investigating tafasitamab monotherapy for patients with r/r DLBCL, 35 patients showed a 12-month PFS rate of 34.3%, with a median duration of response (DoR) of 20.1 months [68]. In a single-arm phase II trial (L-MIND) for the combination of tafasitamab and lenalidomide, 80 non-transplant eligible patients with r/r DLBCL showed a CR rate of 43%, ORR rate of 60%, and DoR of 21.7 months [69]. Given the significant clinical benefits, a phase III trial (NCT02763319) is now recruiting to compare tafasitamab versus rituximab in combination with bendamustine in adult patients with r/r DLBCL. Inebilizumab, a humanized anti-CD19 monoclonal antibody, was tested as monotherapy in a phase 1 study, among which 6 patients with r/r DLBCL were enrolled. The maximum tolerated dose was defined as 8 mg/kg, and ORR was 50% (1 CR and 2 PR) in patients with DLBCL [70].

Coltuximab ravtansine (SAR3419, huB4-DM4) represents a novel CD19-targeted ADC conjugated to a maytansinoid-derivate antimitotic payload DM4 through a disulfide linker. The clinical efficacy and safety of SAR3419 monotherapy were evaluated in a phase II multicenter study. Eighteen of 41 patients with r/r DLBCL at dose 55 mg/m^2^ obtained ORR (43.9%), with a median DoR of 4.7 months [71]. Another CD19-targeted ADC ADCT-402 (loncastuximab tesirine) comprising pyrrolobenzodiazepine dimer toxin showed early promise for patients DLBCL. Of the 51 patients with r/r DLBCL who were treated at 120 mg/kg or above this dosage threshold, 28 (54.9%) responded to ADCT-402, with a median DoR of 3.1 months for patients achieving PR. The DoR for CR patients was not reached with a median follow-up of 7.5 months [72]. Those CD19-targeted ADCs-based combination therapies are under study, which may provide new options for r/r DLBCL.

### CD22-directed agents

Epratuzumab is a CD22-directed monoclonal antibody with efficacy in both relapsed and untreated DLBCL [73]. When combined with rituximab, epratuzumab treatment led to an ORR of 67% and CR of 50% in 6 patients with r/r DLBCL [74]. The 3-year event-free survival (EFS) and OS were 70% and 80%, respectively, in patients with newly diagnosed DLBCL treated with epratuzumab plus standard R-CHOP [75]. After adjusting for IPI, the patients treated with epratuzumab plus R-CHOP achieved significantly improved EFS, compared with those treated with R-CHOP [75]. However, no subsequent follow-up data or phase 3 RCT result was reported, suggesting that no potential extra benefit was provided by addition of epratuzumab to R-CHOP.

Pinatuzumab vedotin (DCDT2980S) is a CD22-directed ADC conjugated to the antimitotic payload MMAE. Pinatuzumab vedotin alone at dose 2.4 mg/kg yielded moderate efficacy, with an ORR of 36% and median DoR of 3.0 months observed in patients with r/r DLBCL [65]. Combination of rituximab and pinatuzumab vedotin resulted in higher ORR and CR in patients with r/r DLBCL, compared with single-agent pinatuzumab vedotin (ORR, 60% vs. 36%; CR, 26% vs. 16%, for combination vs. single agent, respectively) [65]. Inotuzumab ozogamicin (CMC-544) is another CD22-directed ADC conjugated to the DNA-damaging calicheamicin. A phase 1/2 study of combining inotuzumab and rituximab reported an ORR of 74% in r/r DLBCL patients [76], but the phase 3 trial (NCT01232556) of inotuzumab ozogamicin plus rituximab in r/r DLBCL was discontinued for futility in 2013 when comparing with investigator’s choice (IC). However, the favorable safety profiles of inotuzumab plus rituximab suggest this regimen may be appropriate for a specific patient populations [77]. A study of inotuzumab plus rituximab, cyclophosphamide, vincristine, and prednisolone in chemotherapy-naïve patients with DLBCL who are not candidates for anthracycline-based treatment is currently recruiting (NCT01679119).

### CD30-directed agents

Brentuximab vedotin (BV, SGN-35) is a potent CD30-directed ADC, which has been approved by the FDA for classical Hodgkin lymphoma, primary cutaneous anaplastic large cell lymphoma, and systemic anaplastic large-cell lymphoma. The efficacy of BV is under broad investigation in various subtypes of NHL, including DLBCL. CD30 was expressed on 20% or more tumor cells of about 14% of de novo DLBCL cases, though significant association between the response rate and CD30 expression in DLBCL was undefined [78, 79]. Single agent BV was active in r/r DLBCL with variable levels of CD30 expression, and ORR occurred in 44% of DLBCL cases [80]. However, computer-assisted digital image analysis showed that a minimum CD30 expression threshold of 1% was required for antitumor properties in DLBCL [81]. Thus, it is recommended that CD30 immunostaining should be done routinely in DLBCL, and BV may provide a potential option for r/r DLBCL with CD30 positivity.

### CD74-directed agents

CD74 is a MHC class II chaperone broadly expressed on human immune cells and B cell lymphomas, which represents a promising target for treatment of DLBCL [82]. The novel CD74-directed ADC STRO-001 contains a humanized glycosylated antibody SP7219 and potent maytansinoid linker-warhead. STRO-001 is already being investigated in the first-in-human phase 1, multicenter study (NCT03424603) for adults with advanced B cell malignancies, including r/r DLBCL. Preliminary antitumor activity of STRO-001 observed in 4 patients with DLBCL was encouraging. One patient achieved a CR after 2 cycles but progressed after 6 cycles. An additional patient with DLBCL obtained a partial response after 3 cycles [83]. Though modest activity was demonstrated as monotherapy, further trials evaluating the efficacy of combination strategies should be done.

## Molecular pathway inhibitors

**Fig. 4:**
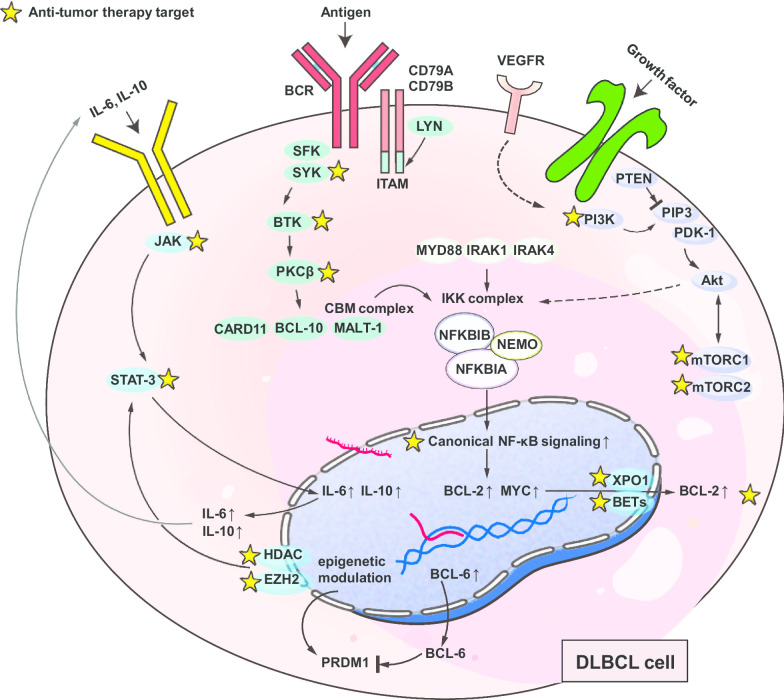
Novel agents targeting molecular signaling pathways and epigenetic regulations. Distinct molecular aberrations classify DLBCL into different molecular subtypes and indicate individualized treatments, including BCR signaling pathways, BCL-2, JAK/STAT3 pathways, VEGFR, PI3K/Akt/mTOR pathways, NF‐κB signaling pathways, as well as epigenetic regulators, such as HDAC, EZH2, and BET

Gene expression profiling analysis has defined ABC and GCB as 2 major subtypes of DLBCL (about 50% and 30%, respectively), according to cell of origin [84]. Aberrant expression and genetic disorders of CD79b, CARD11, MYD88, TNFAIP3, BCL-10, TRAF3, TRAF2, NFKBIA, and NFKBIE (IkBε), in concordance with the prevalence of chronic-active B cell receptor (BCR) signaling, JAK-STAT3 signaling, and canonical NF-κB signaling, were believed to underlie the inferior outcomes of ABC DLBCL (Fig. 4) [85]. Notably, great heterogeneity exists in the entity of GCB or ABC. In 2018, Schmitz and colleagues identified 4 unique genetic subtypes in DLBCL (MCD, BN2, N1, and EZB) with distinct prognosis [85]. At the meantime, Chapuy et al. [86] identified 5 robust DLBCL clusters of discrete outcomes with coordinate genetic signatures. Moreover, George et al. [87] recently developed an algorithm that can classify a patient’s lymphoma into one of seven genetic subtypes, which highlight the potential use of specific targeted agents and contribute to precision medicine. For example, the perturbation of proximal BCR signaling is suggested for MCD subtype, BCL-2 inhibitors for BN2, NF-κB signaling blockade for both BN2 and A53, along with the inhibition of JAK-STAT3 signaling for ST2 subtype [85–87]. In this section, we summarize the cross-linked signaling pathway intricacies at the intersection of DLBCL biology and the clinic. Rational molecular therapies targeting aberrant pathways in the clinical setting are carefully enumerated (Table 4) and discussed on the molecular basis.

**Table 4:** New molecular therapeutics and ongoing clinical trials in diffuse large B cell lymphoma

Agent	Agent type	Combined agent	Study	Study phase	Recruitment status	Enrollment (estimated/actual)	Indication	Results for DLBCL^a^
Idelalisib	PI3K inhibitor	–	NCT03576443 (ILIAD)	2	Recruiting	72	Relapsed GCB DLBCL	–
Copanlisib	PI3Kα/δ inhibitor	Nivolumab	NCT03484819	2	Recruiting	106	DLBCL failing or ineligible for ASCT	–
Parsaclisib	PI3Kδ inhibitor	–	NCT02998476 (CITADEL-202)	2	Active, not recruiting	60	r/r DLBCL	[109]
Parsaclisib	PI3Kδ inhibitor	R-CHOP	NCT04323956	1/1b	Not yet recruiting	44	Newly diagnosed, high-risk DLBCL	–
Parsaclisib	PI3Kδ inhibitor	Rituximab, bendamustine/ibrutinib	NCT03424122	1	Recruiting	81	r/r NHL	–
BR101801	PI3Kδ and DNA-PK dual inhibitor	–	NCT04018248	1	Not yet recruiting	90	Advanced lymphomas	–
Umbralisib (TGR-1202)	PI3Kδ and CK1 dual inhibitor	Ublituximab, bendamustine	NCT02793583 (UNITY-NHL)	2/3	Recruiting	900	Previously treated NHL	–
Everolimus	mTORC1 inhibitor	Lenalidomide	NCT01075321	1/2	Active, not recruiting	58	r/r NHL or HL	–
Temsirolimus	mTORC1 inhibitor	Rituximab, DHAP	NCT01653067	2	Recruiting	88	r/r DLBCL	–
Venetoclax	BCL2 inhibitor	–	NCT01328626	1	Recruiting	222	r/r CLL and NHL	[101]
Venetoclax	BCL2 inhibitor	Lenalidomide, obinutuzumab	NCT02992522	1	Suspended	60	r/r NHL	–
Venetoclax	BCL2 inhibitor	Atezolizumab, obinutuzumab	NCT03276468	2	Recruiting	138	r/r DLBCL and indolent NHL	–
Venetoclax	BCL2 inhibitor	RICE	NCT03064867	1/2	Recruiting	64	r/r DLBCL	–
Venetoclax	BCL2 inhibitor	DA-EPOCH-R	NCT03036904	1	Active, not recruiting	34	DLBCL and HGBCL	–
Venetoclax	BCL2 inhibitor	Obinutuzumab, rituximab, polatuzumab vedotin	NCT02611323	1	Recruiting	134	r/r DLBCL and follicular lymphoma	–
Venetoclax	BCL2 inhibitor	Obinutuzumab	NCT02987400	2	Recruiting	21	r/r DLBCL	–
Entospletinib	Spleen tyrosine kinase inhibitor	R-CHOP	NCT03225924	1/2	Active, not recruiting	25	Newly diagnosed DLBCL aaIPI ≥ 1	–
Ibrutinib	BTK inhibitor	ABT-199, rituximab	NCT03136497	1	Recruiting	30	r/r DLBCL	–
Ibrutinib	BTK inhibitor	ABT-199, prednisone, obinutuzumab, lenalidomide	NCT03223610	1b/2	Recruiting	130	CD20 positive B cell lymphoma	–
Ibrutinib	BTK inhibitor	Loncastuximab tesirine	NCT03684694	1/2	Recruiting	161	Advanced DLBCL, mantle cell lymphoma	–
Ibrutinib	BTK inhibitor	Lenalidomide, rituximab	NCT02077166	1/2	Active, not recruiting	129	r/r non-GCB DLBCL	[119]
Ibrutinib	BTK inhibitor	R-ICE	NCT02955628	2	Recruiting	34	Pre-transplant r/r DLBCL	–
Ibrutinib	BTK inhibitor	Buparlisib	NCT02756247	1	Active, not recruiting	37	r/r DLBCL, FL, mantle cell lymphoma	[95]
ARQ-531	BTK inhibitor	–	NCT03162536	1/2	Recruiting	146	Selected hematologic malignancies	–
LOXO-305	BTK inhibitor	Venetoclax, R-CHOP	NCT03740529	1/2	Recruiting	403	CLL/SLL, NHL	–
DTRMWXHS-12	BTK inhibitor	Everolimus, pomalidomide	NCT04305444	2	Recruiting	120	r/r CLL, NHL	–
Acalabrutinib	BTK inhibitor	–	NCT02112526	1	Recruiting	21	r/r ABC DLBCL	[96]
Acalabrutinib	BTK inhibitor	RICE	NCT03736616	2	Recruiting	47	DLBCL after first-line failure	–
Acalabrutinib	BTK inhibitor	DA-EPOCH	NCT04002947	2	Recruiting	112	untreated DLBCL	–
Acalabrutinib	BTK inhibitor	R-CHOP	NCT03571308	1/2	Recruiting	39	untreated DLBCL	–
Acalabrutinib	BTK inhibitor	pembrolizumab	NCT02362035	1b/2	Active, not recruiting	161	r/r hematologic malignancies	[97]
Enzastaurin	PKCβ inhibitor	R-CHOP	NCT03263026	3	Recruiting	235	untreated DGM1-positive DLBCL, IPI ≥ 3	–
Lenalidomide	Immunomodulatory agent	–	NCT04150328 (RE-MIND)	2	Recruiting	500	r/r DLBCL	[69]
Lenalidomide	Immunomodulatory agent	MOR208	NCT02399085 (L-MIND)	2	Active, not recruiting	81	r/r DLBCL, non-transplant eligible	[69]
Lenalidomide	Immunomodulatory agent	R-CHOP	NCT00670358	1/2	Recruiting	47	Untreated DLBCL	[34]
Lenalidomide	Immunomodulatory agent	R-CHOP	NCT00907348	2	Unknown	49	Elderly untreated DLBCL, IPI ≥ 2	[34]
Lenalidomide	Immunomodulatory agent	R-CHOP	NCT01856192	2	Active, not recruiting	345	Untreated stage II–IV DLBCL	–
Lenalidomide	Immunomodulatory agent	R-CHOP	NCT02285062	3	Active, not recruiting	570	Untreated ABC DLBCL	[36]
Lenalidomide	Immunomodulatory agent	miniCHOP, subcutaneous rituximab	NCT02128061 (SENIOR)	3	active, not recruiting	250	Untreated CD20 + DLBCL, aged over 80 years	[37]
Lenalidomide	Immunomodulatory agent	Rituximab, ibrutinib	NCT02636322	2	Active, not recruiting	60	Newly diagnosed non-GCB DLBCL	[120]
Itacitinib	JAK1 inhibitor	Parsaclisib	NCT02018861 (CITADEL-101)	1/2	Active, not recruiting	88	r/r B cell malignancies	[122]
Itacitinib	JAK1 inhibitor	Ibrutinib	NCT02760485	1/2	Active, not recruiting	33	r/r DLBCL	–
Ruxolitinib	JAK1/2 inhibitor	–	NCT01431209	2	Active, not recruiting	71	r/r NHL failing or ineligible for SCT	[123]
Valemetostat	EZH1/2 dual inhibitor	–	NCT02732275	1	Recruiting	70	Adults with advanced NHL	–
Tazemetostat	EZH2 inhibitor	–	NCT01897571	1/2	Active, not recruiting	420	NHL and advanced solid tumors	[130]
Tazemetostat	EZH2 inhibitor	R-CHOP	NCT02889523	1/2	Suspended	133	Untreated high-risk DLBCL	[132]
Tazemetostat	EZH2 inhibitor	–	NCT03456726	2	Active, not recruiting	21	r/r NHL, EZH2 mutation	–
Tazemetostat	EZH2 inhibitor	–	NCT02875548	2	Recruiting	300	Patients with antecedent tazemetostat study	–
Panobinostat	HDACi	–	NCT01261247	2	Active, not recruiting	41	r/r NHL	–
Vorinostat	HDACi	R-CHOP	NCT00972478	1/2	Active, not recruiting	83	Untreated stage 2–4 DLBCL	–
Chidamide	HDACi	–	NCT03201471	2	Recruiting	39	High-risk DLBCL	–
Romidepsin	HDACi	5-Azacitidine	NCT01998035	1/2	Active, not recruiting	52	r/r NHL	–
Selinexor	XPO1 inhibitor	Venetoclax	NCT03955783	1	Suspended	78	r/r high-risk DLBCL, leukemia	[143]
Selinexor	XPO1 inhibitor	R-CHOP	NCT03147885	1b/2	Recruiting	44	NHL	–
Selinexor	XPO1 inhibitor	RICE	NCT02471911	1	Active, not recruiting	23	r/r aggressive B cell lymphoma	–

*NHL* non-Hodgkin lymphoma, *R-CHOP* rituximab, cyclophosphamide, doxorubicin, vincristine, and prednisone, *DLBCL* diffuse large B cell lymphoma, *r/r* relapsed/refractory, *EZH* enhancer of zeste homolog, *CLL* chronic lymphocytic leukemia, *G-CHOP* obinutuzumab, cyclophosphamide, doxorubicin, vincristine, and prednisone, *RICE* rituximab, ifosfamide, carboplatin, and etoposide, *DA-EPOCH-R* dose-adjusted etoposide, prednisone, vincristine, cyclophosphamide, doxorubicin, and rituximab, *HGBCL* high-grade B cell lymphoma, *HL* Hodgkin lymphoma, *GCB* germinal center B cell like, *PI3K* phosphatidylinositol-3-kinase, *aaIPI* age-adjusted international prognosis index, *BTK* Bruton’s tyrosine kinase inhibitor, *SLL* small lymphocytic lymphoma, *PKCβ* protein kinase Cβ, *PMBCL* primary mediastinal B cell lymphoma, *JAK* janus kinase, *ASCT* autologous stem cell transplantation, *HDACi* histone deacetylase inhibitors

^a^If the study results are published, the reference number will be given

### BCR signaling pathway inhibition

Chronic-active BCR signaling is pivotal in the survival of almost all ABC DLBCLs, driven by frequent activating mutations of the immunoreceptor tyrosine-based activation motifs in CD79B and CD79A, or of the coiled-coil domain in CARD11 [62]. On the contrary, GCB DLBCLs were prone to present with a BCR-negative immunophenotype [88]. Molecular inhibitors targeting BCR-dependent ABC DLBCLs include: entospletinib and fostamatinib for spleen tyrosine kinase (SYK); ibrutinib, zanubrutinib, ARQ-531, LOXO-305, DTRMWXHS-12, and acalabrutinib for BTK; enzastaurin for protein kinase Cβ (PKCβ) (Fig. 4). Of note, SYK inhibitors showed limited single-agent activities in r/r ABC DLBCL [89, 90]. A better understanding of which patients would benefit from BCR blockade via SYK inhibition or other molecular therapeutics is important for their further development in DLBCL. For example, BTK inhibition killed upstream CD79-mutant DLBCL cells, but was dispensable for downstream CARD11-mutant DLBCL cells, which were susceptible to NF-κB pathway inhibitors [91]. Immunohistochemistry and genetic assessments are thereby recommended, in order to confirm the exact lesion in molecular pathways.

Ibrutinib, the first approved BTK inhibitor, has shown activity in the r/r setting of ABC-DLBCLs, especially those with concurrent CD79b and MYD88 mutation [92]. Combined ibrutinib with R-ICE (rituximab, ifosfamide, carboplatin, and etoposide) resulted in an ORR of 90% in r/r DLBCL and a CR rate of 100% in patients with non-GCB subtype [93]. Nevertheless, ibrutinib with R-CHOP did not benefit the overall patients with untreated non-GCB DLBCL in a randomized, placebo-controlled, phase III PHEONIX study [6], but addition of ibrutinib to R-CHOP benefited younger patients of DLBCL, especially those with both c-MYC and BCL-2 overexpression. Since primary resistance to BTK inhibition in DLBCL was associated with BCR signaling activation, ibrutinib combination therapies with venetoclax are now under active clinical investigation [94]. Also, the combination of BTK and PI3K inhibition with ibrutinib and buparlisib was tested, reporting a CR rate of 23% in 13 patients with r/r DLBCL [95]. Besides, acalabrutinib monotherapy showed promising activities among 21 patients with r/r DLBCL, inducing CRs in 5 patients (including 1 GCB DLBCL) [96]. Furthermore, BTK inhibition may synergize with immunotherapy, since acalabrutinib in combination with pembrolizumab resulted in ORRs of 27% in GCB (*n* = 30) and 26% in non-GCB r/r DLBCL (*n* = 31), with a median DoR of 6.9 months [97].

In a randomized phase II trial, frontline PKCβ inhibitor enzastaurin plus R-CHOP showed improved median PFS compared with R-CHOP alone (36 vs. 23 months, respectively), especially for high-risk patients [98]. A new phase III ENGINE study is ongoing to test enzastaurin with R-CHOP in high-risk DLBCL patients positive for DGM1, a genetic biomarker signifying responses to enzastaurin treatment [99].

### BCL-2 inhibition

In DLBCL, B cell lymphoma-2 (BCL-2) overexpression maintained tumor viability through apoptosis inhibition and mediated molecular mechanisms underlying R-CHOP resistance [100]. Constitutive overexpression of BCL-2 was detected in both subtypes of DLBCL through distinct mechanisms: chromosomal translocations in GCB DLBCL and NF-κB signaling activation in ABC DLBCL. *BCL2* translocation was detected in 28.0% of GCB and 0.7% of ABC DLBCL [85]. Therefore, BCL-2 inhibition is most likely to be effective in the cluster 5 with extranodal ABC and cluster 3 with GCB, which exhibited BCL-2 overexpression plus frequent mutations of CD79B and MYD88^L265P^ (Fig. 4), or frequent mutations in epigenetic enzymes (e.g., KMT2D, CREBBP, and EZH2), BCL-2 and PTEN, respectively, as defined by Chapuy et al. [86].

The selective, orally bioavailable BCL-2 inhibitor venetoclax (ABT-199) was tested in a multitude of phase I and II studies. The first-in-human phase I trial of venetoclax reported an ORR of 18% in 34 patients with r/r DLBCL, with an estimated median PFS of 1 month [101]. The most common grade 3 and 4 hematologic adverse events at target doses from 200 to 1200 mg for all NHLs were anemia (15%), neutropenia (11%), and thrombocytopenia (9%) [101]. First-line venetoclax was tested in 56 patients with NHL in combination with R-/G-CHOP, including 18 patients with DLBCL. ORR was reported to be 87.5% for all NHLs, and CR rate was 79.2% and 78.1% in venetoclax with R-CHOP and G-CHOP, respectively [102]. A retrospective cohort study evaluated the off-label use of salvage venetoclax with concomitant therapy in 34 patients with NHL, including 13 DLBCL. With median venetoclax dosed at 400 mg, the ORR was achieved at 26% and CR at 3% in the entire cohort. The observed median PFS for the DLBCL cohort was 2 months [103]. Moreover, preclinical study showed synergistic activity between the BCL-2 inhibitor navitoclax (ABT-263) and bendamustine [104], but the phase II clinical study on navitoclax plus bendamustine and rituximab in r/r DLBCL was withdrawn due to non-safety-related reasons (NCT01423539). Thus, reliable biomarkers need to be extensively investigated to guide the use of BCR inhibition in DLBCL due to the currently modest efficacy.

### VEGFR inhibition

Similar to the fate of bortezomib or ibrutinib in first-line treatment setting of DLBCL, bevacizumab (Avastin), a humanized monoclonal antibody targeting VEGF-A, did not show benefits when added to R-CHOP in patients with newly diagnosed DLBCL [105]. However, this study did not prevent VEGFR from being an effective target in r/r DLBCL. Apatinib is an orally administered novel tyrosine kinase inhibitor targeting vascular endothelial growth factor receptor-2 (VEGFR-2), which involves in lymphomagenesis. Home administration of apatinib with regular outpatient follow-up produced encouraging antitumor effects in r/r DLBCL in an open-label, single-arm, prospective study [106]. ORR of 43.8% and a disease control rate of 71.9% were reported, with a median DoR of 5.0 (95% CI 3.5–6.5) months (*n* = 32). The most common toxicities of any grade were hypertension (62.5%), leukopenia (40.6%), and hand-foot syndrome (40.6%) [106]. The relatively high response rate attained by apatinib deserves future investigation of drug combination strategies.

### PI3K/Akt/mTOR inhibition

PI3K/Akt/mTOR (mammalian target of rapamycin) signaling pathway is involved in the constitutive activation of BCR signaling and cell adhesion-mediated drug resistance within tumor microenvironment [107]. Current clinical results of PI3K/Akt/mTOR signaling inhibition (Fig. 4) showed modest responses in r/r DLBCL. The efficacy and safety of PI3Kα/δ inhibitor copanlisib (Aliqopa; BAY80-6946) were tested in a phase 2 trial, in which patients with ABC r/r DLBCL had an ORR of 13.3%, whereas an ORR of 31.6% was achieved in patients with GCB subtype. The PFS was 1.8 and 4.3 months in ABC and GCB subgroups, respectively. Treatment-emergent adverse events mostly reported were hypertension (40.3%), diarrhea (37.3%), and hyperglycemia (32.8%) [108]. Parsaclisib (INCB050465), a selective next-generation oral PI3Kδ inhibitor, showed single-agent efficacy for r/r DLBCL in a phase 2 trial. ORR was 20% and 25.5%, respectively, for patients who previously received BTK inhibitors or not [109].

Everolimus (RAD001) and temsirolimus (CCI-779) are rapamycin analogues directing against mTORC1. Single-agent everolimus got an ORR of 30% and DoR of 5.7 months in a phase 2 study, which enrolled 77 r/r DLBCL patients after a median of 3 prior therapies. The regimen was well tolerated, and the most common grade 3 and 4 adverse events included thrombocytopenia (38%), neutropenia (18%), and anemia (14%) [110]. Similar outcomes were reported with single-agent temsirolimus in the r/r DLBCL cohort of a phase 2 trial, in which the ORR was 28% with a DoR of 2.4 months [111]. When combined with rituximab, everolimus produced an ORR rate of 38% (9/24) and median DoR of 8.1 months in heavily pretreated DLBCL [112]. Though the phase 3 PILLAR-2 trial reported no significantly improved disease-free survival (DFS) with 1-year everolimus maintenance therapy in poor-risk patients with newly diagnosed DLBCL (hazard ratio, 0.92; 2-year DFS, 77.8% vs. 77.0%, for everolimus vs. placebo, respectively) [113], combined everolimus with R-CHOP-21 produced high EFS12 and EFS24 rates of 100% in the phase 1 Alliance study, in which 96% of newly diagnosed DLBCL patients achieved response [114].

### NF‐κB pathway inhibition

As downstream effector of chronic-active BCR signaling, sustained activity of NF-κB signaling exerts a prominent survival feature for ABC DLBCL. Downstream expressions of cyclinD2, CCR7, IRF4, FLIP, NFKBIA, and BCL-2 were highly expressed in many of the ABC DLBCLs rather than GCB DLBCLs [115]. The proteasome inhibitor bortezomib (Velcade) proves to inhibit NF-κB pathway (Fig. 4) and showed activity in r/r ABC-DLBCL [116]. However, addition of bortezomib to R-CHOP or replacement of vincristine by bortezomib (VR-CAP) did not improve both response rates and long-term survival outcomes in patients with non-GCB DLBCL [4]. It has been demonstrated that functional PRDM1 is required for mantle cell lymphoma response to bortezomib [117], while loss of PRDM1 was found in more than half of the patients with ABC-DLBCL, which may hinder the apoptosis induced by bortezomib [118]. Thus, the expression status of PRDM1 should be assessed before implement of bortezomib in treatment of DLBCL.

Lenalidomide can also exert direct tumor toxicities via binding to cereblon to inhibit downstream NF-κB signaling [33]. As aforementioned, lenalidomide has been shown to provide benefits for r/r ABC-DLBCL [39, 40], as well as elderly patients when used as maintenance therapy [35]. Moreover, promising and durable activity was observed for triplet ibrutinib, rituximab, and 10–25 mg lenalidomide (IR2 regimen) in r/r DLBCL, particularly in non-GCB DLBCL (ORR: 65% vs. 29%; median DoR: 15.9 vs. 8.8 months, for non-GCB vs. GCB, respectively) [119]. In SMART START trial, the same triplet combination with 25 mg lenalidomide as a leading-in regimen in the first-line setting for non-GCB DLBCL gave impressive results, with an ORR and CR rate of 86% and 36%, respectively, after two cycles of IR2 treatment [120]. Prolongation of IR2 use and reduction in chemotherapy cycles are needed in future exploration, especially for those relatively unfit patients.

### JAK/STAT3 inhibition

STAT3 expression was detected in 37% of DLBCL and 54% of ABC DLBCL and signified poor survival especially for the ABC subtype when treated with R-CHOP [121]. Conceivably, activation of the JAK/STAT3 signaling pathway in ABC DLBCL indicates promising therapeutic targets, including JAK, STAT3, and IL-10 receptor (Fig. 4). JAK inhibitors, such as the JAK1 inhibitor itacitinib (INCB039110) and JAK1/2 inhibitor ruxolitinib, have been investigated for the treatment of r/r DLBCL, with phase I/II results already reported. Itacitinib 300 mg once daily was tested in the CITADEL-101 study combined with parsaclisib, but all 6 patients with r/r DLBCL had best overall response of progressive (metabolic) disease [122]. Ruxolitinib produced a median PFS of 1.8 months and OS of 5 months in r/r DLBCL who were ineligible for, or failed SCT (*n* = 32) [123]. According to results from a phase Ib trial, AZD9150, a next-generation antisense oligonucleotide inhibitor of STAT3 mRNA showed efficacy in patients with r/r DLBCL [124]. Two in 27 patients achieved CRs (1 each at 2 mg/kg and 3 mg/kg dose levels), and 2 achieved PRs, announcing a median DoR of 10.7 months [124]. From the data shown above, JAK/STAT3 inhibition seems unworthy of further investigation in DLBCL, unless predictive biomarkers are available to guide treatment with this strategy.

### Selective inhibitors of nuclear export

The selective inhibitors of nuclear transport (SINE) have been developed as a novel class of anti-DLBCL agents [125]. The most well-known SINE inhibitor is selinexor (KPT-330, XPOVIO), which is a first-in-class, investigational oral therapeutic that selectively blocks exportin 1 (XPO1) and leads to reductions in *MYC* and *BCL2* oncogenes (Fig. 4) [126]. Selinexor has demonstrated notable efficacy in the open-label SADAL phase IIb study [126] and received final approval from FDA for the treatment of patients with r/r DLBCL after at least 2 lines of systemic therapy in June 2020. Among this SADAL population of 127 patients, selinexor produced an ORR of 28% and CR of 12%, with a median DoR of 9.3 months. In the cohort with prior SCT, the greatest benefits were observed (ORR: 44%; median PFS: 5.9 months). Most common grade 3–4 adverse events were thrombocytopenia, neutropenia, and anemia [126]. It is worth exploring XPO1 inhibitor-based combinational therapy in r/r setting after R-CHOP failure according to the above impressive findings, but special attention should be paid to the severe adverse events when using selinexor.

## Epigenetic-modifying drugs

Epigenetic modulation, such as DNA methylation and histone deacetylation, involves in tumorigenesis among lots of solid tumors and hematologic malignancies. Increasing data have demonstrated both direct antitumor activity and enhancement of the function of immune cells, making it an appealing strategy in the treatment of DLBCL (Fig. 4).

### Histone deacetylase inhibitors

Histone deacetylase inhibitors (HDACis), including panobinostat, vorinostat (Zolinza, SAHA), chidamide (HBI-8000), and romidepsin (FR901228), are used as novel, off-label anticancer epigenetic therapies for DLBCL. At 30 mg three times weekly, panobinostat resulted in an ORR of 28% in patients with r/r DLBCL in a phase II trial, with a median DoR of 14.5 months [127]. Although the response rate was not impressive when using as monotherapy, those who got remission enjoyed a relatively long duration of remission. Thus, useful biomarkers to predict patients who are deemed to benefit from panobinostat are urgently needed. Rituximab combination did not increase responses, while MEF2B mutations and circulation tumor DNA (ctDNA) reduction were predictors of early responses [127]. Mondello et al. demonstrated in vitro that panobinostat induced mutations in the STAT3 binding site to downregulate mutant-MYD88 transcription, inhibited NF-κB activation, and promoted ibrutinib efficacy in ABC DLBCL cells [128]. This provides foundation for the combination therapy with ibrutinib and panobinostat in ABC DLBCL, especially for the cluster 5 defined by Chapuy et al. [86]. Because HDACi exhibited immunomodulatory effects and could synergize with immune checkpoint inhibitors to produce enhanced antitumor activity, vorinostat and pembrolizumab demonstrated an ORR of 56% and a CR of 33% in r/r DLBCL (*n* = 9) [129].

### EZH2 inhibition

Enhancer of zeste homolog 2 (EZH2) is a histone methyltransferase, repressing nuclear transcription by trimethylating histone H3 lysine 27. 22.0% of GCB and 1.7% of ABC DLBCL exhibited gain-of-function mutations in EZH2 that mediated epigenetic modification and led to tumor survival [85]. Tazemetostat (EPZ-6438) is an oral, first-in-class, selective small-molecule EZH2 inhibitor, which has been approved by FDA to treat adult patients with relapsed or refractory follicular lymphoma (FL) whose tumors are positive for an EZH2 mutation and who have received at least 2 prior systemic therapies, or those who have no optimal alternative treatment options. Single-agent efficacy of tazemetostat in NHL and advanced solid tumors was studied in a first-in-human phase I/II trial [130, 131]. Interim results from the phase II trial showed an ORR rate of 40% in DLBCL with EZH2 mutations and 18% in DLBCL without mutations [131]. Other trials of tazemetostat in DLBCL indications include a phase I/II study evaluating tazemetostat in combination with R-CHOP for high-risk newly diagnosed DLBCL patients. Phase Ib of this study determined 800 mg as the recommended phase 2 dose [132]. Preliminary efficacy data were encouraging with a metabolic CR rate of 76.5% (13/17), and the duration of CR was 2–14 months [132]. Long-term safety and overall survival of patients treated with tazemetostat will be evaluated in the rollover study TRuST (NCT02875548). Other EZH2 inhibitors, such as CPI-1205 [133] and GSK2816126 [134], have also shown promising anti-DLBCL activity and tolerable safety profiles in preliminary phase 1 studies. MAK683, the embryonic ectoderm development protein (EED) inhibitor, can induce reduced tumor cell proliferation in EZH2 mutated cells through binding to EED to block the interaction between EED and EZH2. A phase 1/2 study is undergoing to evaluate the efficacy of MAK683 in a variety of malignancies, including DLBCL (NCT02900651). Further, the EZH1 and EZH2 dual inhibitor valemetostat (DS-3201b) had antitumor activities in both ABC and GCB DLBCL cells in vitro, which is now under investigation in a phase I trial for advanced NHL including DLBCL [135].

### Bromodomain inhibitors

Bromodomain inhibitors are a novel generation of small -molecule inhibitors targeting BET (bromodomain and extra terminal) proteins, which normally trigger gene transcription via complicated mechanisms. Some oncogenes are under epigenetic modulations by BET, such as c-MYC [136]. Thus, bromodomain inhibitors may present with antitumor efficacy by suppressing the expression of those oncogenes. OTX015 (MK8628), a selective inhibitor of BET, showed prominent anti-lymphoma activity in vitro [137]. Preclinical investigations demonstrated that OTX015 had various targets, including NF-KB/TLR/JAK/STAT signaling pathways, MYC-related genes, and genes that regulate cell cycle [137]. In a phase I trial, 37 patients (including 18 DLBCL patients) were treated with OTX015 monotherapy, and unsatisfactory efficacy was observed with one CR and one PR in patients with heavily pretreated DLBCL. The prognosis for patients with r/r double-hit lymphoma (concurrent *BCL2* and *MYC* translocations) is extremely poor without active salvage agents. Based on the results of in vitro study, BET inhibitors alone or in combination with BCL-2 inhibitors may provide therapeutic potential for patients with MYC-dependent lymphomas in the future [138].

## Conclusion

**Fig. 5:**
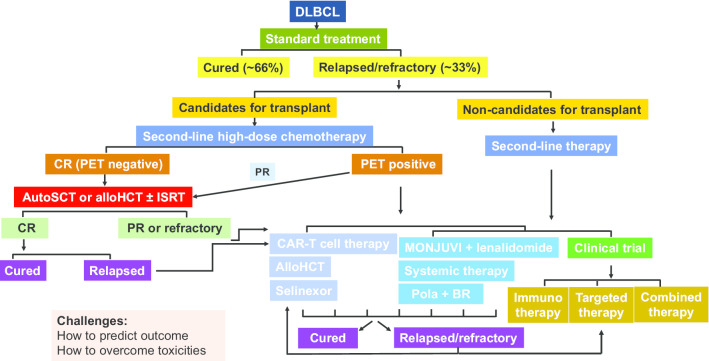
Recommended treatment for DLBCL. *PET* positron emission tomography and computed tomography, *SCR* stem cell rescue, *ISRT* involved site radiotherapy, *CAR* chimeric antigen receptor, *HCT* stem cell transplantation, *Pola + BR* polatuzumab vedotin combined with bendamustine and rituximab

Due to the great heterogeneity of DLBCL, one-third of patients will eventually failed R-CHOP treatment, and great challenges exist regarding how to accurately predict outcomes and provide individualized salvage therapies (Fig. 5) [144]. Although several novel molecular subtyping systems have been developed those years, about half of the patients could not be classified into a specific subtype, and there is still a long way to go before implementation of those molecular subtypes in routine clinical practice. From the data shown above, CAR-based cell therapies exhibit the most promising results. Multi-target CAR T cells, combination of different mono-target CAR T cells, CAR T cells combined with ICIs or novel molecular inhibitors, or fourth-generation CAR T cells with safety switches can further improve both the efficacy and safety profiles in r/r DLBCL. Similarly, different target-based BiTEs are also promising due to convenient accessibility. Regarding the small molecular inhibitors or epigenetic modifying drugs, it is impossible to cure DLBCL with monotherapy because no driver gene aberrations have been identified for DLBCL. However, with so many new drugs in the development pipeline, there will be enormous number of drug combination mode, which is extremely difficult to investigate in clinical trials due to limited patient resources. Exploring reliable biomarkers to guide individualized treatment is worth many efforts, and screening drugs with potential synergistic effect is helpful to design combinational trials. Moreover, the potential superimposed toxicity profiles should be considered when novel drugs with distinct mechanisms of action are used together, especially for DLBCL where many targets are not specific and off-target effects are inevitable. Meanwhile, unlike cytotoxic drugs, many novel targeted agents or immunotherapies work slowly in patients, and pseudo-progress occurs at some point, which warrants up-to-date response criteria. Finally, though a long way toward the cure of DLBCL, with the guidance of detailed genetic information, the optimal combination of both novel and traditional drugs will emerge to promote precision medicine in patients with DLBCL.

## Data Availability

The datasets supporting the conclusions of this study are included in the figures and tables.
